# Systematic Review and Meta-analysis of Postexposure Prophylaxis for Crimean-Congo Hemorrhagic Fever Virus among Healthcare Workers

**DOI:** 10.3201/eid2409.171709

**Published:** 2018-09

**Authors:** Önder Ergönül, Şiran Keske, Melis Gökçe Çeldir, İlayda Arjen Kara, Natalia Pshenichnaya, Gulzhan Abuova, Lucille Blumberg, Mehmet Gönen

**Affiliations:** Koç University, Istanbul, Turkey (Ö. Ergönül, M.G. Çeldir, İ.A. Kara, M. Gönen);; American Hospital, Istanbul (Ş. Keske); Rostov State Medical University, Rostov-on-Don, Russia (N. Pshenichnaya);; South-Kazakhstan State Pharmaceutical Academy, Shymkent, Kazakhstan (G. Abuova);; National Institute for Communicable Diseases, Johannesburg, South Africa (L. Blumberg)

**Keywords:** Crimean-Congo hemorrhagic fever, ribavirin, healthcare workers, postexposure prophylaxis, early treatment, CCHF, viral hemorrhagic fever, PRISMA, meta-analysis, viruses, vector-borne infections, zoonoses, systematic review, Crimean-Congo hemorrhagic fever virus, CCHFV, Turkey, Pakistan, Germany, Iran, India, South Africa, Russia, Tajikistan, Arabic Emirates, Mauritania, Kazakhstan, Sudan, Albania, and Spain

## Abstract

We performed a systematic review and meta-analysis on the effectiveness of ribavirin use for the prevention of infection and death of healthcare workers exposed to patients with Crimean-Congo hemorrhagic fever virus (CCHFV) infection. Splashes with blood or bodily fluids (odds ratio [OR] 4.2), being a nurse or physician (OR 2.1), and treating patients who died from CCHFV infection (OR 3.8) were associated with healthcare workers acquiring CCHFV infection; 7% of the workers who received postexposure prophylaxis (PEP) with ribavirin and 89% of those who did not became infected. PEP with ribavirin reduced the odds of infection (OR 0.01, 95% CI 0–0.03), and ribavirin use <48 hours after symptom onset reduced the odds of death (OR 0.03, 95% CI 0–0.58). The odds of death increased 2.4-fold every day without ribavirin treatment. Ribavirin should be recommended as PEP and early treatment for workers at medium-to-high risk for CCHFV infection.

Crimean-Congo hemorrhagic fever (CCHF) virus (CCHFV) is listed as a highly infectious pathogen that could cause a public health emergency (http://www.who.int/medicines/ebola-treatment/WHO-list-of-top-emerging-diseases/en/). CCHFV infection has been reported from >30 countries in Africa, Asia, Europe, and the Middle East (*1*,*2*). CCHFV is a member of the genus *Orthonairovirus* in the family *Nairoviridae* that causes severe disease in humans; the reported case fatality rate is 3%–30% (*1*). Humans can become infected through the bites of ticks, by contact with patient blood or bodily fluids, or by contact with blood or tissues from viremic livestock. The risk for human-to-human transmission of CCHFV increases in parallel with the lack of preparedness (*3*).

Healthcare workers need to be well prepared against the emerging threat of CCHF outbreaks. The efficacy of postexposure prophylaxis (PEP) with ribavirin for high-risk exposures to CCHFV needs clear evidence. The relatively low secondary attack rates of CCHFV and ethics constraints make controlled, prospective efficacy trials for ribavirin PEP unlikely. In the absence of efficacy studies, a thorough examination and logical extrapolation of the existing data can be useful for developing recommendations. The efficacy of PEP for healthcare workers exposed to CCHF patients might be similar to that for healthcare workers with high-risk exposures to Lassa fever patients (*4*). A series of cases of healthcare workers infected with CCHFV has been reported (*5*–*10*). Integration of the details on the exposures and the outcomes of the infections from these published reports could provide the opportunity to generate powerful conclusions about the risk for infection and death among healthcare workers. We described the efficacy of PEP with ribavirin for CCHFV infection and early ribavirin use in CCHF treatment.

## Methods

### Search Strategy

We performed a systematic review of individual participant data (IPD) and reported data by using PRISMA-IPD (Preferred Reporting Items for Systematic Reviews and Meta-Analyses for IPD) guidelines (*11*). We searched PubMed, Google, ProMED, and conference proceedings by using the keywords “Crimea-Congo hemorrhagic fever,” “health care worker,” “nosocomial,” “CCHF,” and “health professional.” We included all published reports in peer-reviewed journals through September 3, 2017.

### Definitions and Outcome of Interest

We defined CCHFV exposure as visible contact or imperceptible contact (i.e., contact with patient aerosols) with a CCHF patient. Primary outcomes were infection with no symptoms, infection with symptoms, and death. The primary study objective was to assess the protective role of PEP and early ribavirin treatment. Early treatment was defined as treatment occurring <48 hours after the onset of symptoms.

### Exposure Risk Groups

Healthcare workers were grouped into 3 categories with respect to their risks for CCHFV infection. The high-risk group consisted of healthcare workers who were directly exposed to blood or bodily fluids of a CCHFV-infected patient, such as through a needle stick or splash. Healthcare workers in this group were categorized as being without personal protective equipment (PPE) of any sort by default because a PPE breach had occurred. The moderate-risk group comprised healthcare workers without a known direct exposure to blood or bodily fluids of a CCHFV-infected patient but either handled patients who bleed or visibly produced other body fluids or were involved in aerosol-producing procedures (e.g., intubation, bronchoscopy, and resuscitation) without wearing an N95 mask. The low- or unknown-risk group consisted of healthcare workers who cared for CCHFV-infected patients who did not actively bleed or produce bodily fluids and did not participate in aerosol-producing procedures.

### Inclusion and Exclusion Criteria

In this study, we included healthcare workers who were exposed to CCHFV through a defined transmission event who did and did not receive PEP, healthcare workers with laboratory-confirmed CCHFV infections who had a detailed exposure history and were closely followed by laboratory tests for their clinical outcomes, and symptomatic healthcare workers who did and did not receive ribavirin <48 hours after the onset of symptoms. In the reports from Albania (*12*,*13*), Pakistan (*14*,*15*), South Africa (*9*,*16*,*17*), and India (*18*,*19*), some of the cases were duplicates (included in >1 article). In these instances, we avoided duplicated data and selected the case information from the earlier publication for inclusion. We did not include seroprevalence studies, gray literature, or screening reports for tracing cases that did not have defined exposures; if the information was incomplete or lacking, we requested the information directly from the authors, and we did not include the articles if the data we needed were unavailable.

### Data Collection

We entered IPD obtained from reports into a structured data sheet. We performed analyses using an integrated dataset in Stata version 11 (https://www.stata.com/). In our dataset, we included information on demographics, transmission, PEP, the course of infection, the number of days from onset of disease, and treatment. The dataset also included information on the predictors of infection and death. Study authors (Ö.E., Ş.K., M.G.Ç., and İ.A.K.) resolved discrepancies during discussions with local physicians.

### Statistical Analysis

We followed the PRISMA-IPD statement guidelines (*11*) using R studio (https://www.rstudio.com/). We used a 2-stage approach for analyses. First, we analyzed the studies that were suitable for calculating an effect estimate (odds ratio [OR]). Then, we pooled all the participant studies, including single case reports, and calculated a common effect estimate (OR) and 95% CI. We used random effects models.

### Bias Assessment

We performed an analysis for confounders with the integrated dataset. We used the χ^2^ test for categorical data and *t*-test for continuous data and performed logistic regression to detect potential confounding predictors of infection and death. To predict infection, we included in our model the covariates PEP with ribavirin, being in the high-risk group, being a nurse or physician, and providing care for a CCHF patient who died. To predict death, we included in our model the covariates days from onset of symptoms to ribavirin treatment, being in the high-risk group, and being a nurse or physician. These were the most critical variables for predicting death. In statistical analyses, we used Stata version 11, and we considered p values <0.05 statistically significant. For meta-analysis, we used meta: General Package for Meta-Analysis version 4.9-1 (https://cran.r-project.org/web/packages/meta/index.html).

## Results

**Figure 1:**
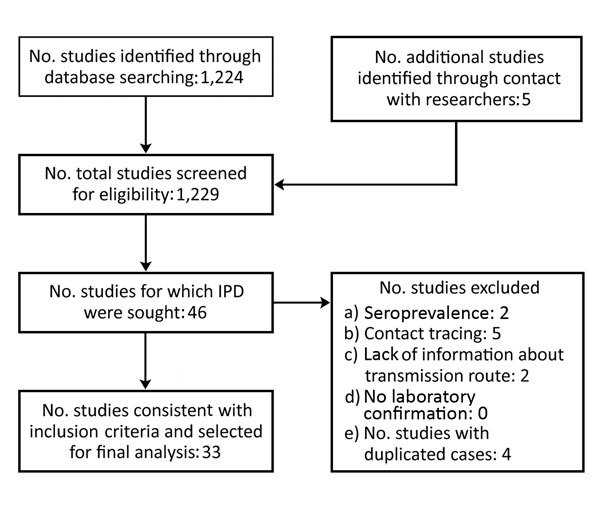
Identification and selection of studies included in a meta-analysis of the effectiveness of postexposure prophylaxis with ribavirin and early treatment with ribavirin among healthcare workers exposed to patients infected with Crimean-Congo hemorrhagic fever virus, 1976–2017. IPD, individual participant data.

We reviewed 1,224 published reports on CCHF, and 33 studies met our inclusion criteria (Figure 1). In the included studies, 175 healthcare workers from Turkey (*5*,*7*,*20*–*25*), Pakistan (*15*,*26*–*32*), Germany (*6*), Iran (*33*–*36*), India (*18*,*19*), South Africa (*9*), Russia (*8*), Tajikistan (*37*), United Arab Emirates (*38*), Mauritania (*39*), Kazakhstan (*40*), Sudan (*41*,*42*), Albania (*12*), and Spain (*2*) were exposed to patients infected with CCHFV (Table). We included all of the healthcare workers who were infected, but because of the lack of detailed exposure histories among those who were not infected, we excluded 47 healthcare workers from Tajikistan (*37*), 75 from Turkey (*5*,*10*), 40 from Germany (*6*), and 33 from Pakistan (*15*,*30*). The diagnoses were based on reverse transcription PCR results for 58 (57%) healthcare workers, ELISA for 47 (46%), both ELISA and reverse transcription PCR for 26 (25%), immunofluorescence assay for 13 (12%), and complement fixation for 10 (10%).

**Table:** Characteristics and outcomes for healthcare workers exposed to patients with Crimean-Congo hemorrhagic fever virus infection, 1976–2017

Country (references)	No. (%)					
	Exposed, N = 175	High risk, n = 107	Moderate risk, n = 65	Low or no known risk, n = 3	Infected, n = 102	Died, n = 34
Turkey (*5**,**7**,**20**–**25*)	49 (28)	23 (22)	26 (40)	0	19 (19)	3 (9)
Pakistan (*15**,**26**–**32*)	45 (26)	21 (20)	24 (36)	0	18 (18)	6 (18)
Germany (*6*)	18 (10)	18 (17)	0	0	2 (2)	0
Iran (*33**–**36*)	12 (7)	10 (9)	1 (2)	1 (33)	12 (12)	3 (9)
India (*18**,**19*)	8 (5)	5 (5)	3 (5)	0	8 (8)	6 (18)
Russia (*8*)	8 (5)	6 (6)	0	2 (67)	8 (8)	0
South Africa (*9*)	8 (5)	3 (3)	5 (8)	0	8 (8)	2 (6)
Tajikistan (*37*)	7 (4)	7 (7)	0	0	7 (7)	2 (6)
United Arab Emirates (*38*)	5 (3)	1 (1)	4 (6)	0	5 (5)	2 (6)
Kazakhstan (*40*)	5 (3)	3 (3)	2 (3)	0	5 (5)	3 (9)
Mauritania (*39*)	5 (3)	5 (5)	0	0	5 (5)	5 (15)
Sudan (*41**,**42*)	3 (2)	2 (2)	1 (2)	0	3 (3)	2 (6)
Albania (*12*)	1 (1)	1 (1)	0	0	1 (1)	0
Spain (*2*)	1 (1)	(1)	0	0	1 (1)	0

The population of healthcare workers included in our study was 53% male and 47% female. The percentages of infected male and female healthcare workers did not differ (p = 0.828), and the percentage of symptomatic male (38%) and female (29%) healthcare workers who died was not significantly different (p = 0.413). Among symptomatic healthcare workers, the mean age was 33 (SD 8.5, range 20–61) years, and the case-fatality rate was 34%. The percentage of symptomatic cases did not differ from the percentage of asymptomatic cases (p = 0.545), and the case-fatality rate was not higher in symptomatic than asymptomatic healthcare workers (p = 0.674). Being a nurse or physician (p = 0.01) and caring for a CCHF patient who died (94% of infected healthcare workers vs. 80% of noninfected healthcare workers; p = 0.006) were factors more common among healthcare workers who were infected than those who were not.

**Figure 2:**
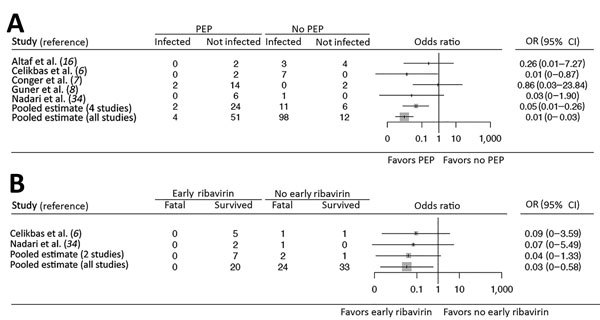
Effectiveness of PEP and early treatment with ribavirin among healthcare workers exposed to patients infected with Crimean-Congo hemorrhagic fever virus, 1976–2017. A) Two-step meta-analysis of the effectiveness of PEP with ribavirin for preventing Crimean-Congo hemorrhagic fever virus infection. We could determine the effect estimates for only 4 individual studies, and we included 33 reports in the final pooled estimate. B) Two-step meta-analysis on the effectiveness of early ribavirin use for preventing death caused by Crimean-Congo hemorrhagic fever virus infection. We could determine the effect estimate for only 2 individual studies, and we included 33 reports in the final pooled estimate. OR, odds ratio; PEP, postexposure prophylaxis.

We performed 2 meta-analyses: 1 on the effectiveness of PEP with ribavirin to prevent CCHFV infection (Figure 2, panel A) and 1 on the effectiveness of early ribavirin treatment after CCHF symptom onset to prevent death (Figure 2, panel B). In the first stage of the meta-analysis on PEP, the OR could be calculated for only 4 studies (OR 0.05, 95% CI 0.01–0.26); at the second stage, after pooling all healthcare worker study data, the OR was 0.01 (95% CI 0–0.03; Figure 2, panel A). The heterogeneity of these studies was not significant (I^2^ = 3%, Γ^2^ = 0.1157; p = 0.38). During the first stage of the meta-analysis on early ribavirin use, the OR could be calculated for only 2 studies (OR 0.04, 95% CI 0–1.33); at the second stage of the analysis, after pooling all healthcare worker study data, the OR was 0.03 (95% CI 0–0.58; Figure 2, panel B). No heterogeneity was detected among these studies (I^2^ = 0%, Γ^2^ = 0; p = 0.92).

In univariate analyses of healthcare workers exposed to CCHF patients, splashes with blood or bodily fluids (OR 4.2, 95% CI 2.04–9.7; p<0.001), being a nurse or physician (OR 2.1, 95% CI 1.13–4.1; p = 0.019), and caring for a patient who died (OR 3.8, 95% CI 1.38–10.46; p = 0.01) significantly increased the odds of a healthcare worker acquiring an infection. However, PEP with ribavirin significantly reduced the risk for CCHFV infection (OR 0.01, 95% CI 0.003–0.03; p<0.001). To control for confounders, we performed a multivariate analysis of the data set. In multivariate analyses of exposed healthcare workers, PEP with ribavirin was found to significantly protect against CCHFV infection (OR 0.009, 95% CI 0.001–0.039; p<0.001). In a sensitivity analysis, the area under the receiver operating curve was 94%. In a multivariate analysis of symptomatic healthcare workers adjusted by risk group (high risk vs. others) and worker type (nurse or physician vs. others), the risk for death increased 2.4-fold for every day of delay in the start of ribavirin treatment (OR 2.4, 95% CI 1.27–4.56; p = 0.005). Appropriate use of PPE and PEP with ribavirin predicted death completely; therefore, both of these factors were not included in the model. The sensitivity of this model, calculated by the area under the receiver operating curve, was 95%.

**Figure 3:**
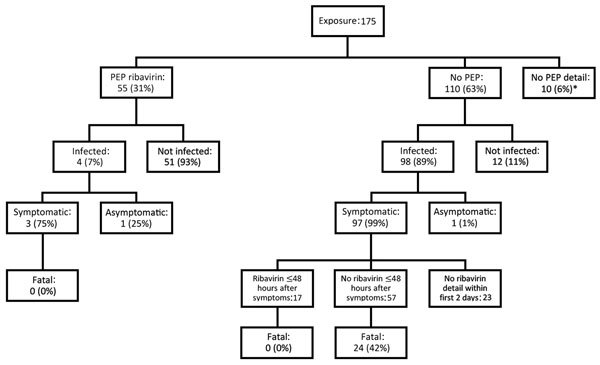
Flowchart of healthcare workers exposed to patients infected with Crimean-Congo hemorrhagic fever virus who did and did not receive PEP with ribavirin or early ribavirin treatment <48 hours after symptom onset, 1976–2017. *Healthcare workers for which PEP information was not included in the original report. PEP, postexposure prophylaxis.

Of 175 healthcare workers exposed to CCHF patients, 55 (31%) received and 110 (63%) did not receive PEP with ribavirin (Figure 3). In the PEP arm, 7% acquired infection, and in the no PEP arm, 89% acquired infection (p<0.001; Figure 3). In the no PEP arm, 97 (99%) of 98 infected healthcare workers became symptomatic. None of the symptomatic healthcare workers who received ribavirin within 48 hours after the onset of symptoms died, whereas 42% of the symptomatic healthcare workers who did not receive ribavirin within 48 hours died (p<0.001; Figure 3). Among symptomatic healthcare workers who received ribavirin, the median time from onset of symptoms to ribavirin treatment was 5 days for those who died and 1.25 days for those who survived (p<0.001).

For 104 (59.4%) of 175 healthcare workers, the appropriateness of the PPE was assessed by the authors of the original report. The percentage who became infected was lower for those who used PPE appropriately (55%) than those who did not (70%), although this difference was not significant (p = 0.301). No fatal cases were reported among those who used PPE appropriately. In all reports, the PPE used included a mask, gloves, and a gown; in 1 study in Germany (*6*), the additional use of goggles was reported.

## Discussion

We analyzed all published reports on healthcare workers who were exposed to CCHF patients and had a moderate-to-high risk of acquiring a CCHFV infection. These cases represent the case density in the 14 countries included, in parallel with previous reports (*43*,*44*). Most cases were from Turkey, Pakistan, and Iran. However, healthcare workers could acquire the infection from persons from other countries (*6*), and in 2017, a nurse in Spain acquired (*2*) a CCHFV infection from a patient with an autochthonous case.

We determined that PEP with ribavirin reduced CCHFV infection among healthcare workers and early ribavirin use reduced death among CCHFV-infected healthcare workers (Figure 2). In most case series and case reports, no healthcare workers died who received PEP with ribavirin (*5*–*8*,*10*), including those who received PEP soon after a high-risk incident (Figure 3).

Early use of ribavirin in the treatment of CCHFV infection has been reported as beneficial (*45*,*46*) and is considered to be beneficial, despite a controversial report (*47*). In the report in which authors disagreed with ribavirin use being beneficial, the authors did not account for the starting time of ribavirin treatment after symptom onset, even though this factor can significantly alter its efficacy. Close follow-up of infected healthcare workers provides an opportunity to determine the efficacy of early ribavirin use; assessing the quality of treatments given to exposed healthcare workers is much more feasible than assessing that of patients with suspected CCHF transferred from rural areas. In this study, we showed that every day of treatment delay increases the risk for death by 2.4-fold. Of note, none of the symptomatic healthcare workers who received ribavirin within 48 hours after the onset of symptoms died, whereas 42% of those who did not receive treatment in that time frame died (p<0.001; Figure 3). Late diagnosis of the source case can result in delayed PEP and treatment of healthcare workers with ribavirin (*21*,*30*).

Some centers have reported aerosol CCHFV transmission (*5*–*8*,*33*,*48*). A number of procedures (e.g., bronchoscopy, nasal tamponade, intubation, cardiopulmonary resuscitation) as well as patient bleeding can lead to the aerosolization of CCHFV. Persons near CCHFV patients during these activities should be considered at moderate risk for acquiring the infection. Awareness of transmission after percutaneous injuries is high, but healthcare workers with imperceptible exposures to aerosolized pathogens should also be considered for close follow-up. Our study findings indicate that PEP with ribavirin should be recommended for those with CCHFV exposures, similar to the recommendations for healthcare workers with Lassa virus exposures (*4*).

In this study, we included all published reports of detailed, laboratory-confirmed cases; we avoided duplicated cases and excluded screenings of healthcare workers with nonspecified risk (*5*,*6*,*15*,*28*,*49*,*50*). One limitation of this study is reporting bias; we did not include unreported cases. Because of medical and legal issues, some fatal cases involving healthcare workers who were not using PPE appropriately or who did not receive PEP might not have been reported. For example, 2 fatal cases involving healthcare workers who were not given ribavirin, 1 from Turkey (http://www.hurriyet.com.tr/gundem/kan-alirken-eline-igne-batan-kubra-kkka-dan-oldu-11861967) and 1 from Pakistan (https://www.samaa.tv/uncategorized/2016/07/congo-fever-cases-emerge-in-bahawalpur/), were not published in the literature, but their stories appeared in the media. Even though we received detailed information about these cases, we did not include them in our study because they were not published in peer-reviewed journals. These unreported fatal cases support the use of ribavirin for PEP and early treatment, as we recommend in this report.

Another limitation of this study was regarding the reporting of the appropriateness of the PPE used, which was reported for only ≈60% of the healthcare workers included. PPE use is not standardized; appropriate use varies substantially from country to country. For instance, in a study in Germany, the use of surgical masks instead of N95 masks during aerosol-generating procedures is considered inappropriate (*6*); however, this practice was considered appropriate in many other studies. Implementing standard use of PPE in healthcare settings is urgently needed. Our study shows that N95 masks should be used in high- and moderate-risk events, including those involving contact with patients who are bleeding or visibly generating bodily fluids or aerosols.

Our analyses were performed by using an integrated dataset that included all necessary detailed information about the demographics, transmission, PEP, course of the infection, days from onset of disease, and treatment. This dataset could be supplied to researchers in the field and used as a tool for future investigations.

In conclusion, our results indicate a significant beneficial effect of PEP with ribavirin after CCHFV exposure. This beneficial effect extended to early use of ribavirin for treatment of infected healthcare workers. Imperceptible contact with infectious particles and splashes of blood or bodily fluids from infected patients should all be considered and prevented. A universal standard of care that includes PPE and PEP and treatment with ribavirin should be implemented for healthcare workers at risk for CCHF.
